# Structure, Morphology and Reducibility of Epitaxial Cerium Oxide Ultrathin Films and Nanostructures

**DOI:** 10.3390/ma8095278

**Published:** 2015-08-31

**Authors:** Paola Luches, Sergio Valeri

**Affiliations:** 1 Istituto Nanoscienze, Consiglio Nazionale delle Ricerche, Via G. Campi 213/a, Modena 41125, Italy; 2 Dipartimento di Scienze Fisiche Informatiche e Matematiche, Università degli Studi di Modena e Reggio Emilia, Via G. Campi 213/a, Modena 41125, Italy; E-Mail: sergio.valeri@unimore.it

**Keywords:** reducible oxides, reactive molecular beam epitaxy, scanning tunneling microscopy, X-ray photoelectron spectroscopy, low-energy electron diffraction, interface structure, surface morphology, strain, oxidation state

## Abstract

Cerium oxide is a very interesting material that finds applications in many different fields, such as catalysis, energy conversion, and biomedicine. An interesting approach to unravel the complexity of real systems and obtain an improved understanding of cerium oxide-based materials is represented by the study of model systems in the form of epitaxial ultrathin films or nanostructures supported on single crystalline substrates. These materials often show interesting novel properties, induced by spatial confinement and by the interaction with the supporting substrate, and their understanding requires the use of advanced experimental techniques combined with computational modeling. Recent experimental and theoretical studies performed within this field are examined and discussed here, with emphasis on the new perspectives introduced in view of the optimization of cerium oxide-based materials for application in different fields.

## 1. Introduction

Cerium oxide is a subject of intense research in several different fields of materials science. One of its peculiarities is that Ce cations can easily and reversibly switch between two oxidation states, a Ce^4+^ and a Ce^3+^ state, leading to the possibility of rapidly forming, filling, and moving oxygen vacancies within the material [1]. Among all the applications of cerium oxide-based materials, the most successful one is represented by car catalytic converters, where a cerium-zirconium mixed oxide is used as a support for platinum group metal nanoparticles [2]. Promising prospective applications of cerium oxide-based materials include catalysts for hydrogen production through the water-gas shift reaction [3], proton-exchange membrane fuel cell electrodes [4], and electrolytes for solid oxide fuel cells in combination with other oxides [5]. 

A very large number of recent studies point to a deeper understanding of different aspects of cerium oxide-based materials. Among them, an interesting research line focuses on ultrathin films and nanostructures epitaxially supported on single crystalline substrates. These model systems often show interesting novel properties, induced by spatial confinement and/or by the interaction with the supporting substrate. In some cases, metastable structural phases without a bulk counterpart can be stabilized at different values of the oxygen chemical potential, and they can be reversibly transformed, one into the other, by means of oxidizing and reducing treatments [6,7,8,9]. The atomic scale understanding of such systems often implies the use of state-of-the-art experimental techniques with high spatial and chemical resolution and high sensitivity, coupled with advanced theoretical modeling [10,11]. The outcomes of these studies are very relevant in view of the design of materials with optimized properties in the different fields of applications.

The use of metallic substrates allows the application of electron spectroscopies and scanning tunneling microscopy (STM); however, in some cases, insulating or semiconducting supports and different characterization techniques have also been used [9,12,13,14]. The model systems are typically grown in ultrahigh vacuum by reactive molecular beam epitaxy with various preparation procedures. In the earliest studies, cerium oxide films were obtained by the deposition of metallic cerium followed by post-oxidation [15]. Subsequent investigations showed that films with a better structural ordering could be obtained by oxidizing surface alloys of Ce and substrate (Pt or Ru) atoms, in turn formed by depositing metallic Ce on the substrate at high temperatures or by post-growth annealing of a metallic Ce film [16,17]. More recent studies use the reactive growth of cerium in oxygen atmosphere, followed by a post-growth thermal treatment—also in oxygen atmosphere—to optimize the film stoichiometry, structure, and morphology [17,18,19]. Accurate studies of the influence of the growth parameters on the film properties allowed researchers to obtain systems with tunable stoichiometry and size of morphological surface features [6,20,21,22]. Typically, above approximately 1000–1100 K, depending on the substrate used, the oxide films showed to be thermally unstable and they reconverted into complex surface alloys [6,9,15].

The aim of the present review is to examine and discuss selected recent studies dealing with ultrathin epitaxial cerium oxide films and nanostructures, with emphasis on the novel properties induced by reduced dimensionality and/or by the proximity of a metallic substrate.

This review is organized as follows: Section 2 focuses on the epitaxy of the films on different substrates, discussing issues related to the structural strain and to the interfacial charge configuration; Section 3 focuses on the oxidation state of the films at different thicknesses and preparation conditions and on the different morphologies observed; and Section 4 discusses the phases obtained when epitaxial films are deliberately reduced.

## 2. The Interface between Cerium Oxide and Metal Surfaces: Epitaxy, Strain, and Charge Transfer

Bulk cerium oxide has the fluorite structure, in which the (111) surface—made of alternating oxygen-cerium-oxygen trilayers—is the most stable one [23]. This repeating unit will be referred to as a monolayer (ML) in the following. Most of the studies of cerium oxide epitaxial films have been dealing with the stabilization of the most stable (111) surface orientation. Some studies, however, have also been focused on the growth of films exposing less stable surfaces and on the understanding of the mechanisms for their stabilization [13,24,25].

The different metal substrates used for epitaxial growth of cerium oxide induce, in some cases, relevantly different properties in the ultrathin films due to the specific interfacial atomic and electronic structure. In some cases, the different preparation conditions used can be partially responsible for some of the observed modifications, but it is clear that in many cases the film/metal interaction is very important in determining the overall behavior of the films. 

**Figure 1:**
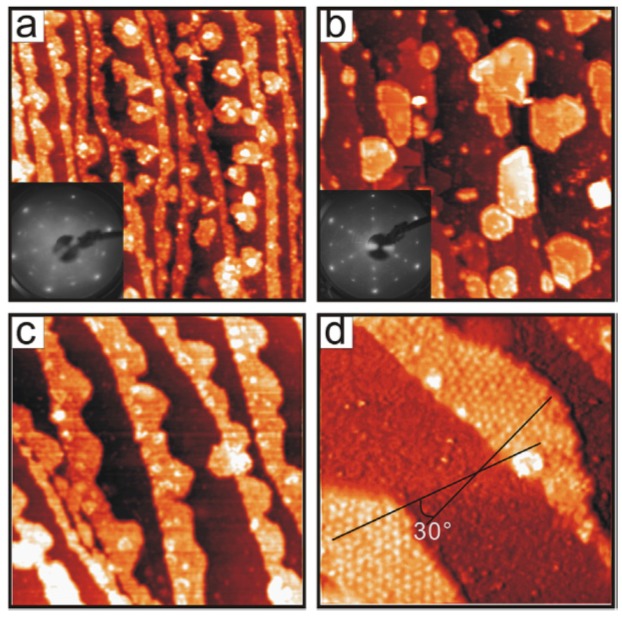
STM images of 0.6 ML cerium oxide film deposited on Ru(0001) at 700 K (**a**) and 790 K (**c**) in 1 × 10^−7^ mbar of O_2_; (**b**) shows sample (**a**) further annealed in 4 × 10^−7^ mbar O_2_ at 900 K. Image (**d**) is a close-up of the image (**c**), where two domains exhibiting the moiré structure with a 19 Å periodicity are rotated by 30° with respect to each other. Insets in (**a**) and (**b**) show the corresponding LEED patterns. Image size is 200 × 200 nm^2^ (**a**)–(**c**) and 60 × 60 nm^2^ (**d**). Reprinted with permission from Lu *et al.* [18]. Copyright Elsevier 2006.

The cerium oxide (111) surface shows a six-fold symmetry and it is terminated by a layer of oxygen atoms. The cerium oxide lattice parameter, amounting to 3.83 Å on the (111) surface, is significantly larger than the one of most 3*d* and 4*d* metals by as much as 30%–40%. In spite of this huge lattice mismatch, epitaxial films exposing the (111) surface have been obtained on a number of metal substrates with hexagonal surface symmetry, such as Pt(111) [15,16,17,19], Cu(111) [20,26], Rh(111) [6], Ru(0001) [18,27], Au(111) [28], and many more. On most of these substrates, the cerium oxide films grow with a preferential (111) orientation and with the in-plane symmetry directions aligned with the substrate ones. This occurs due to the formation of substrate/overlayer coincidence supercells made by *n* cerium oxide surface unit cells matching *m* substrate surface unit cells, which represent the translational repeating unit, hereafter named the *n:m* coincidence. This specific epitaxy is often more stable than the formation of polycrystalline films with (111) surface orientation and random in-plane orientation and it is typically stabilized by the post-growth thermal treatment. For example, the Cu(111) surface lattice parameter is almost exactly two-thirds smaller than the cerium oxide one and a 2:3 coincidence cell has been shown to be established [10], while on the Ru(0001) surface, a 5:7 coincidence cell is observed [18,22]. The formation of coincidence cells can be observed in STM images and in low energy electron diffraction (LEED) patterns. An example is shown in Figure 1, reporting STM and LEED images acquired on a CeO_2_ film of sub-monolayer coverage grown on a Ru(0001) surface [18]. As shown in Figure 1d, ultrathin cerium oxide films on Ru(0001) form rotational domains and have a 19 Å surface periodicity, compatible with a different contrast at specific sites of the (5 × 5) coincidence supercell. Rotational domains have been observed not only in the case of Ru(0001) [18,29], but also on other substrates, such as Pt(111) [19], Rh(111) [6], Cu(111) [10], and Re(0001) [30]. The stability of a specific rotation angle for the domain is determined by the cerium oxide substrate interaction. 

**Figure 2:**
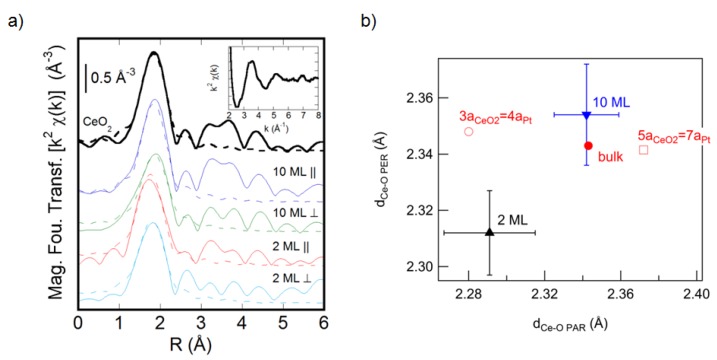
(**a**) Modulus of the Fourier transform of the k^2^-weighted Ce L_3_ edge χ(k) (solid lines) and first shell fits (dashed lines) for the CeO_2_ reference sample and for 10 ML and 2 ML CeO_2_ films on Pt(111) with the electric field parallel and perpendicular to the film surface. The inset shows the Ce L_3_ edge raw χ(k) data for the 10 ML film. (**b**) Ce-O interatomic distances parallel (d_Ce-O PAR_) and perpendicular (d_Ce-O PER_) to the film surface for a 2 ML (black triangle) and for a 10 ML (blue triangle) cerium oxide film grown on Pt(111), obtained from the fitting of the spectra in (**a**). The experimental values are compared with the values measured on the CeO_2_ reference sample (red dot) and with the values expected under the hypotheses of 3:4 (red open circle) and 5:7 (red open square) coincidence, assuming the bulk elastic constants. Reprinted from Luches *et al.* [31] with permission. Copyright ACS 2011.

It is interesting to note that films with a thickness of a few ML tend to adopt a different structure than thicker ones, due to stronger epitaxial and/or dimensionality effects. For example, a polarization-dependent extended X-ray absorption fine structure (EXAFS) study of cerium oxide films on Pt(111) has clearly shown a dependence of the in-plane and out-of-plane lattice parameters on film thickness (Figure 2) [31]. Films with a thickness of the order of 2 ML have an in-plane compressive strain, compatible with the 3:4 coincidence, as shown in Figure 2b [31]. Interestingly, the out-of-plane lattice parameter is different from the one expected using the bulk elastic constants, probably due to a structural rearrangement related to reduced dimensionality [31]. Thicker films are found to relax to the bulk structure in the in-plane and out-of-plane Ce-O distances (Figure 2) [31]. 

A more recent study allowed a direct investigation of the CeO_2_/Pt interface by aberration-corrected scanning transmission electron microscopy measurements (STEM) on a cross-sectional lamella. The study showed that in thicker films, the interface coincidence is maintained, though it locally varies from 3:4 to 2:3 and 5:7 to accommodate the strain [32]. Even on the Cu(111) surface, where the epitaxial mismatch in the *2:3* coincidence is negligible, low thickness films have been observed to have an in-plane compression with respect to thicker ones [10]. The compressive strain was ascribed in this case to a reduced dimensionality effect, which dominates over the epitaxial constraint [10]. The idea that, at reduced dimensionality, the film structure and properties are different than on thicker ones is further confirmed by theoretical calculations. A density functional theory (DFT) study of a single-layer cerium oxide film on Pt(111) substrate showed a significant corrugation of the oxide film and of the metal substrate [33]. The interface electronic configuration was shown to be dominated by charge rearrangements due to electrostatic interactions, but some charge transfer from the substrate to the oxide was also detected [33]. On 3 ML thick films, a different absorption geometry was found to be the most stable, as confirmed by STEM measurements [32]. 

A very strong rearrangement of the interface atomic and electronic structure has been detected at the cerium oxide/Cu interface, where the formation of a (2 × 2) supercell of oxygen vacancies has been detected, leading to the full reduction of the Ce ions at the interface to the Ce^3+^ oxidation state [10]. A measurable, though incomplete, reduction has been measured by spatially resolved electron energy loss also for the cerium ions at the interface with the Pt(111) surface [32]. The charge transfer, probably influenced by the metal work function, which is lower for the copper substrate, certainly also modifies other properties in the film, such as its reducibility and reactivity. 

**Figure 3:**
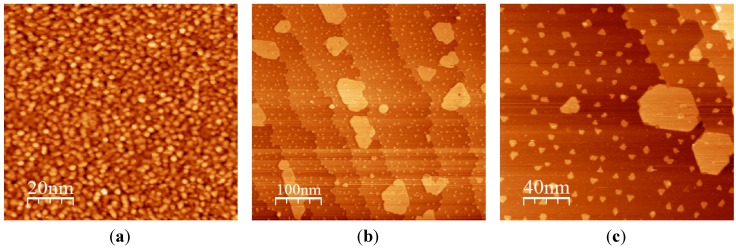
STM images showing the morphology of cerium oxide films on Pt(111): (**a**) 2 ML film after reactive growth at room temperature; (**b**) 0.2 ML film after reactive growth at room temperature and post-growth thermal treatment in O_2_ at 1040 K; (**c**) zoom of sample (**b**). Modified with permission from Luches *et al.* [19]. Copyright ACS 2011.

More complex phases can also be formed at the interface between the cerium oxide and the substrate on which it is grown. For example, platinum oxide islands were formed at the interface between cerium oxide and Pt (small triangular islands in Figure 3a,b), and they have been suggested to mediate the cerium oxide epitaxial growth [19]. On the Cu(111) surface, the formation of a Cu oxide phase has instead been shown to favor the formation of (100)-oriented CeO_2_ nanostructures [34]. On the Au(111) substrate, on the contrary, the formation of Au-Ce alloys has been shown to prevent the formation of extended and well-ordered cerium oxide films [28]. It has to be mentioned for completeness that, in some cases, the substrate-cerium interactions may also prevent the epitaxial growth, such as in the case of Si, where complex cerium silicate phases have been shown to be formed at the interface even at very high values of the oxygen chemical potential [12,35] and routes to limit their formation, including Si surface passivation [14], are necessary to obtain good quality epitaxial films. 

## 3. Oxidation State and Morphology of CeO_2-*x*_ Epitaxial Films

The stoichiometry of cerium oxide films is typically investigated by analyzing the Ce 3*d* XPS spectrum, which results from contributions of cerium ions in the 3+ and 4+ oxidation state. Ce 3*d* XPS spectra can be resolved by a well-established fitting procedure, obtaining the Ce^3+^ and Ce^4+^ concentration within the XPS probing depth [36,37]. Indeed, other XPS lines, like the Ce 4 *d* or even the O 1*s*, also contain contributions from different oxidation states [22], though the fitting procedure contains more ambiguities. The use of synchrotron radiation allows us to tune the photon energy around the Ce 4*d* → 4*f* resonance, increasing the sensitivity to the different oxidation states and decreasing the probing depth [6,38]. 

The earliest works on metal-supported cerium oxide films already pointed out that in the ML thickness regime, a non-negligible Ce^3+^ concentration could be detected, while only thicker films showed the CeO_2_ stoichiometry [6,39,40]. The non-negligible fraction of Ce ions reduced to the 3+ state can be ascribed to a higher density of low coordination sites at low thickness, and to the lower energy for oxygen vacancy formation at those sites. Moreover, the charge transfer from the metal substrate to the oxide, discussed in Section 2, can also induce a measurable reduction of the interface layers, whose weight increases with reducing film thickness. The use of strongly oxidizing gases, such as atomic oxygen [17,19], has been shown to decrease the Ce^3+^ concentration in the films, although at low coverage, the films are rarely in the fully oxidized state [19]. A preferential localization of the Ce^3+^ sites at the interface with Rh(111) was clearly observed by comparison of XPS and resonant photoemission spectra of the valence band [6], demonstrating that the charge transfer is likely to have a non-negligible role.

Through accurate control of the growth procedures, the stoichiometry and the morphology of the films can be finely tuned, obtaining control of the number of open layers and the surface step density, as shown by the Matolin group using Cu(111) as a substrate [21]. A recent work on the Ru(001) substrate clearly shows that the best morphology in terms of the terrace width and the sharpness of the terrace step edges can be obtained by reactive deposition at temperatures close to the onset temperature for film reduction [22]. In ultrathin films on Pt(111), the post-growth thermal treatments in O_2_ at high temperatures (700–1100 K) induce an agglomeration and a flattening of the cerium oxide features observed after growth at room temperature, leading to the formation of islands with definite shape and size, as shown in Figure 3. The islands are typically quasi-hexagonal (Figure 3b,c) and show straight edges, a few tens of nm long, aligned along the 〈110〉 symmetry directions. The quasi-hexagonal shape corresponds to a comparable stability of the two-step edge orientations. The energy of the specific facets exposed by the steps has been evaluated theoretically and compared to the experimental images measured by STM on cerium oxide films on Ru(0001), allowing us to identify the experimental conditions for the stabilization of the different kinds of steps [41]. In specific cases, depending on the substrate used, particularly for the Cu(111) substrate [10] or for the Ru(0001) substrate [22], triangular islands can also be observed. The minimum height of the islands also varies, and only in some cases can the single-layer islands be obtained [6,17,18], while in other cases, the minimum thickness corresponds to approximately 2 ML [19] or more [22].

Interestingly, in some cases, rectangular islands tens of nm in height have been observed both after prolonged heating in vacuum [24] and after re-oxidation of previously reduced films in O_2_ pressure, on Ru(0001) and Pt(111) substrates, respectively [7]. Nilius *et al.* have assigned the rectangular features to (100)-oriented nanostructures and they identified, by a comparison with DFT calculations, the mechanism for the stabilization of the polar surface in the formation of two different surface reconstructions, namely (2 × 2) and c (2 × 2) [24]. As previously mentioned, rectangular (100)-oriented cerium oxide islands were also stabilized on the Cu(111) surface [25,34]. Stesovych *et al.* identified three-dimensional CeO_2_(001) features, which form on top of rectangular cerium oxide interfacial layers, and they observed a (2 × 2) and a (√2 × √2) surface reconstruction, which probably corresponded to oxygen vacancies that partially compensate for the surface dipole moment [25]. Yang *et al.* could isolate ultrathin flat islands without surface reconstruction on the same substrate and they identified a different mechanism for polarity compensation which involves the sharing of an oxygen layer among the cerium oxide and the interfacial copper oxide [34].

**Figure 4:**
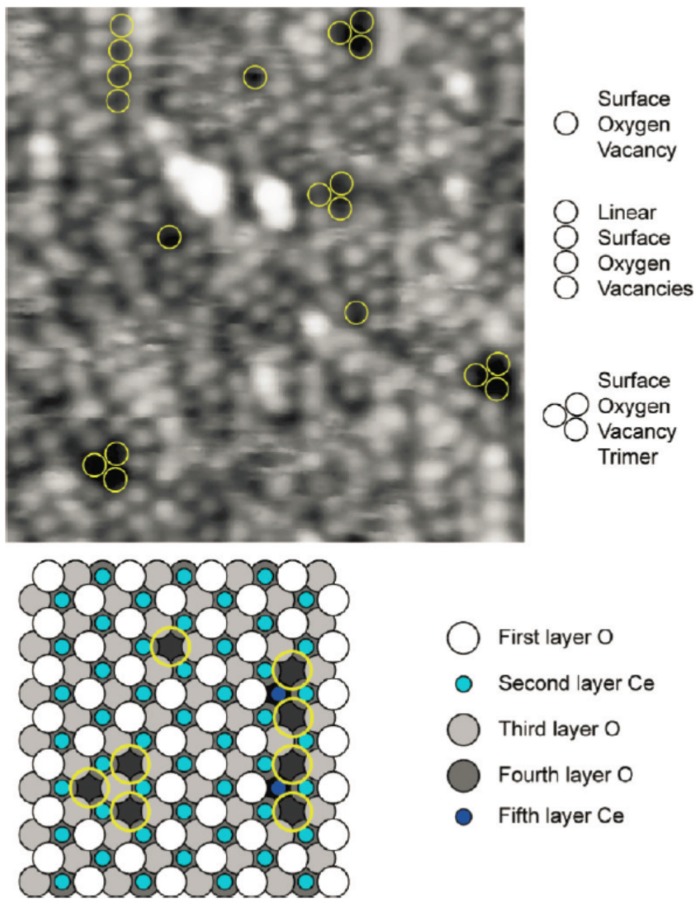
Atomically resolved, filled state, STM image, and structural model depicting surface oxygen vacancies on an ultrathin film of CeO_2_ on Pt(111). Bright spots correspond to top-layer oxygen termination of the surface, and surface vacancies are observed as isolated individuals, trimers, and linear arrangements, as highlighted by yellow circles superimposed on the STM image. Reprinted with permission from Grinter *et al.* [17]. Copyright ACS 2010.

Expectedly, the morphological and structural quality of the surface of thicker films does not depend much on the specific substrate used, but rather on the post-growth oxidizing thermal treatments performed after the growth. On atomically flat film surfaces, the most typical defects observed are comparable to those formed on the (111) surface of cerium oxide single crystals. Though the proper assignment of the features observed by STM is still under debate, most of the studies have agreed on the formation of surface and subsurface oxygen vacancies [17,18,19], and of surface vacancy clusters [17] on the flat areas of cerium oxide terraces (see Figure 4).

## 4. Reduced Cerium Oxide Epitaxial Films

A very relevant aspect is the possibility to investigate the reduction process on cerium oxide ultrathin films in the form of model systems and to create specific reduced phases, which can be re-oxidized to the original CeO_2_ stoichiometry. In this way, it is possible to target the functionality of cerium oxide in proximity with metals in real systems, where catalytic processes involve the release and uptake of oxygen from the material. The most stable cerium oxide-reduced phase in the bulk is the A-type Ce_2_O_3_ phase, with an orthorhombic structure. Other structural polymorphs with Ce ions in the 3+ oxidation state can also be stabilized under specific conditions. Among them, the C-type bixbyite Ce_2_O_3_ phase is particularly interesting, since it is derived from the cubic fluorite structure by removal of one quarter of the oxygen ions in an ordered way after a slight structural rearrangement. The possibility for cerium oxide to be easily and reversibly reduced is, in fact, linked to the stability of a high concentration of oxygen vacancies within the fluorite structure, giving rise to a number of intermediate metastable phases between the fluorite and the C-type bixbyite phase [42]. 

Cerium oxide epitaxial films can be reduced by different treatments, like thermal treatments in vacuum [6,7,19,21], ion bombardment [43], or exposure to reducing gases such as CO [44], methanol [45], or H_2_ [43] at elevated temperatures. Indeed, it is also possible to grow films at reduced oxygen pressures to obtain CeO_2-*x*_ films with 0 < *x* < 1/2 [27,29,46]. 

**Figure 5:**
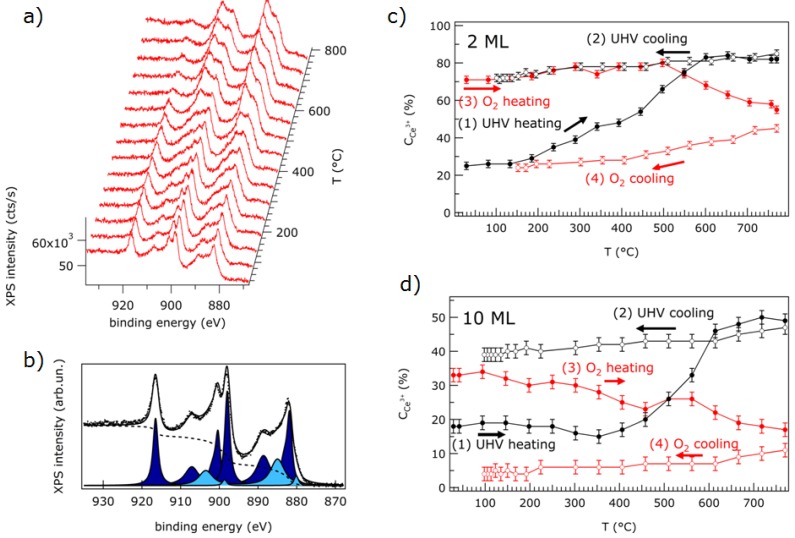
(**a**) Ce 3*d* XPS spectra for a 2 ML cerium oxide film on Pt(111) measured at increasing heating temperature in ultra high vacuum (UHV); (**b**) Ce 3*d* XPS spectrum of a 2 ML film as prepared (dots), fit (solid line), Shirley-type background (dashed line), and components ascribed to Ce^3+^ (light blue) and Ce^4+^ (dark blue) ions; (**c**) Ce^3+^ concentration obtained by fitting the Ce 3*d* XPS spectra of a 2 ML thick film during thermal cycles in UHV and in O_2_ partial pressure; (**d**) Ce^3+^ concentration obtained by fitting the Ce 3*d* XPS spectra of a 10 ML thick film during thermal cycles in UHV and in O_2_ partial pressure. Reproduced from Ref [7] with permission from the PCCP Owner Societies.

Using thermal treatments in vacuum, the reduction starts at the surface and the concentration of Ce^3+^, as evaluated from the analysis of XPS data, is indeed much higher on lower thickness films (Figure 5) [7]. However, an absolute quantification of the Ce^3+^ concentration in the samples is difficult, since the depth profile of Ce^3+^ ion concentration is, in general, unknown. It was shown that ultrathin films start showing a non-negligible concentration of oxygen vacancies at lower temperatures compared to thicker films (Figure 5) [7]. This effect can be induced by reduced dimensionality and to the consequently higher concentration of low coordination sites, but the influence of the metal substrate should also be considered. The possible interfacial charge transfer may, in fact, play a role in decreasing surface oxygen vacancy formation energy in ultrathin films compared to thicker films, where the surface-interface distance is much larger and does not significantly modify the electronic properties of surface sites at which reduction starts. 

A dependence of the temperature at which surface reduction starts on the growth temperature has been demonstrated for cerium oxide films on Cu(111), and it has been related to the density of reduced coordination sites [21]. Furthermore, it has been demonstrated that in cerium oxide, the dynamic processes occurring during reduction, namely oxygen vacancy formation, oxygen transport from subsurface layers, and possible structural rearrangements, are quite slow, so the final degree of reduction is not only determined by the final temperature reached, but also by the heating rate and heating time [7].

**Figure 6:**
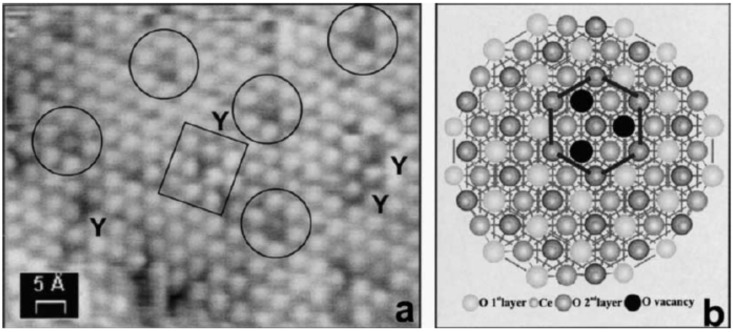
(**a**) High-resolution STM image of the surface of a CeO_2-*x*_ island on Rh(111), showing atomic-scale details of the triple oxygen vacancy defects forming a superlattice (circles) and of Y-shaped defects of a different kind (rectangle). (**b**) Model of the triangular defect in terms of three missing O atoms. Reprinted with permission from Castellarin-Cudia *et al.* [47]. Copyright Elsevier 2004.

A clear indication that the substrate plays a role in the oxygen vacancy formation is given by the observation of ordered arrays of surface oxygen vacancies on the surface of a mildly reduced cerium oxide thin film on the Rh(111) surface, as shown in Figure 6 [47]. The individual defect features have been ascribed to triple oxygen vacancies [47]. The oxygen vacancy network has a periodicity which corresponds to the *5:7* coincidence supercell, and its formation is explained by hypothesizing that due to substrate-overlayer interaction, some sites of the supercell may have a lower energy for oxygen vacancy formation [47]. This effect can be ascribed to structural inhomogeneities within the cell, such as the presence of a variable local stress in the different sites [47] and/or to a different local charge configuration.

Reduced cerium oxide films on other substrates, such as Pt(111), instead show a corrugated surface morphology with apparent clusters ascribed either to electronic modifications [7] or to cerium ions adsorbed on oxygen vacancies [22]. 

**Figure 7:**
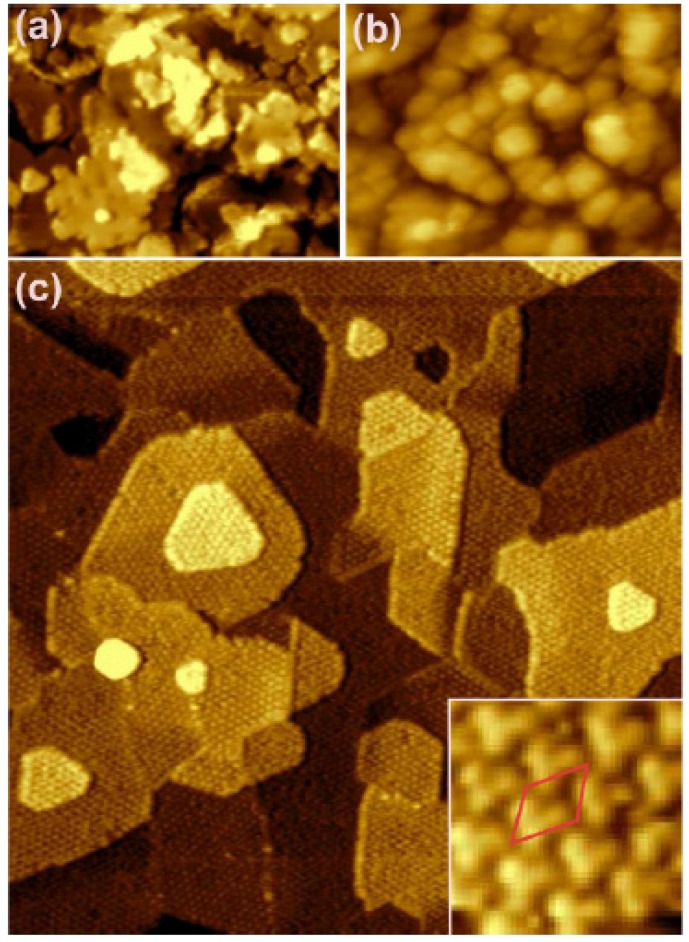
STM images showing the different stages of formation of c-Ce_2_O_3_ films on Cu(111). (**a**) CeO_2_ buffer, (**b**) CeO_2_ buffer with subsequent metallic Ce deposition, (**c**) ordered c-Ce_2_O_3_ layer obtained by annealing (b) in vacuum at 900 K. Inset: High-resolution image and surface unit cell (red rhombus) of the c-Ce_2_O_3_ layer. Images a–c are to scale. Image width (**a**,**b**) 60 nm, (**c**) 120 nm, (inset) 6 × 6 nm^2^. Reprinted with permission from Stetsovych *et al.* [49]. Copyright ACS 2013.

The structural evolution during reduction also presents an interesting aspect investigated mainly by LEED and low energy electron microscopy (LEEM). Phases with peculiar surface reconstruction have been observed both on thin films on metals [7,8], and on thicker films on silicon [9,48]. In general, up to a certain degree of reduction, the films show a LEED pattern which is substantially unaltered compared to the one of the CeO_2_ phase [6,7,27]. This probably indicates that the reduction proceeds by the formation of oxygen vacancies at random sites on the film surface or subsurface, without a detectable ordering or a significant structural rearrangement. Only at higher degrees of reduction has clear evidence for C-type Ce_2_O_3_ structure stabilization been obtained using the Cu(111) [49] and the Si(111) substrates [9,14]. In the first case, the authors used a complex preparation procedure involving the deposition of metallic Ce on a CeO_2_ buffer layer, followed by 900 K annealing [49]. The surface morphology after the three different preparation steps is shown in Figure 7. The C-type structure is identified by a sharp (4 × 4) LEED pattern, compatible with the ordering of O vacancies, by a (4 × 4) surface periodicity in the STM (Figure 7c) and by a complete reduction as observed by the analysis of Ce 3*d* XPS spectra [14,49]. 

**Figure 8:**
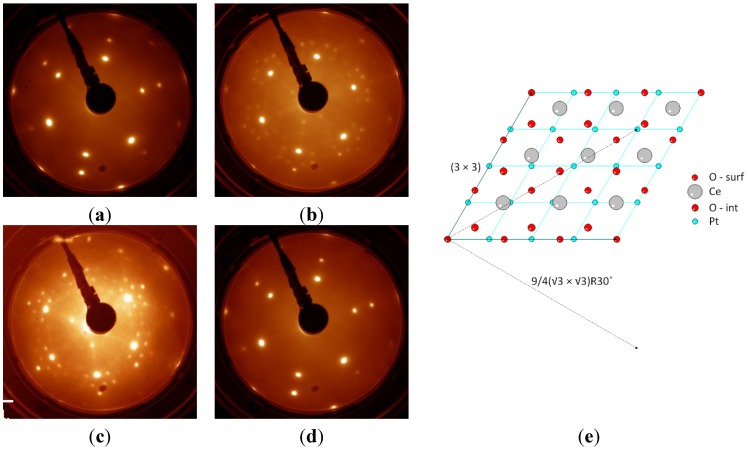
LEED patterns (*E* = 80 eV) of a 2 ML cerium oxide film on Pt(111) (**a**) as prepared, (**b**) after intermediate reduction by heating in UHV at 770 K for 30 min (c_Ce3+_ ~ 40%, as measured by XPS), showing the (3 × 3) and 9/4(√3 × √3)R30° phase (**c**) after strong reduction by heating in UHV at 1040 K for 15 min (c_Ce3+_ ~ 60%–80%) and showing the 9/4(√3 × √3)R30° phase (**d**) after re-oxidation by heating in O_2_ at 1040 K. (**e**) Model of the 3:4 coincidence cell at the interface between CeO_2_ and Pt(111), showing evidence of the vectors of the (3 × 3) supercell (solid line) and of the 9/4(√3 × √3)R30° supercell (dashed lines), which coincide with specific atoms in the coincidence cell. Atoms labeled by O-surf and O-int represent surface and interface oxygen layers, respectively. Adapted from Ref [7] with permission from the PCCP Owner Societies.

At lower degrees of surface reduction, structures with surface periodicity not compatible with any of the observed bulk phases have been stabilized by reduction. For example, on the Pt(111) substrate, after UHV heating at 1040 K, (3 × 3) and 9/4(√3 × √3) LEED patterns have been observed in a wide range of reducing conditions, though only when the film thickness was limited to a few ML [7]. Having excluded the formation of Pt-Ce-O surface alloys, we believe that the observed phase is stabilized by the presence of the substrate, since the observed periodicities coincide with specific sites within the coincidence supercell (Figure 8), and since it was never observed on the surface of thicker cerium oxide films exposed to comparable reducing treatments [7].

The (3 × 3) LEED pattern has also been observed on films grown on the Cu(111) substrate at intermediate degrees of surface reduction and it is ascribed to a specific microscopic model which represents a bulk termination of the CeO_1.67_ phase [8]. Other surface reconstructions are common to different substrates and milder reduction states. Among these, the (√7 × √7)R19.1° was originally stabilized on Si(111) [48], and subsequently observed also on Cu(111) [8]. In most cases, the LEED reconstruction was ascribed to periodicities arising from ordered oxygen vacancies, although only in the (4 × 4) Ce_2_O_3_ specific case was it possible also to directly image the vacancies by STM [8]. 

The different phases stabilized in reduced cerium oxide films at reduced dimensionality have also been addressed by theoretical studies employing the simulated mechanical annealing approach to select the most stable structures, which were subsequently simulated by DFT [50]. The study shows that the relative stability of the C-type and A-type phases in ultrathin films depends on the in-plane lattice parameter [50]. Furthermore, it identifies a new structure without a bulk counterpart which is more stable than the A-type and C-type phases at larger in-plane lattice parameters, such as those that might be induced by epitaxy on Re(0001) or Pt(111) [50]. This and related studies provide important input to unravel the origin of the complex phases identified experimentally and to understand their properties. 

An important point is also the reversibility of the reduction process, which has been demonstrated in some cases on thermally reduced ultrathin films [7,8]. On the contrary, if a Si(111) substrate without passivation is used, a thermal treatment similar to the one that gave reduced cerium oxide films on Pt gives origin to a fully but irreversibly reduced phase ascribed to a cerium silicate [12]. 

## 5. Conclusions

This work reviews recent studies of ultrathin epitaxial cerium oxide films, which allowed us to obtain important insights on the properties of this material at reduced dimensionality and on its complexity. The work done until now represents an important input for studies concerning the reactivity of catalysts made of metal nanoparticles supported on cerium oxide and of model inverse catalysts made of cerium oxide epitaxial nanostructures on metal supports. 

Efforts are still required to face important issues such as the understanding of metastable structural phases in the form of ultrathin films and the stabilization of nanostructures exposing less stable surfaces. Other research directions, currently at an early stage, though promising for the future, also include the possibility to increase the complexity of the investigated epitaxial systems, for example, by introducing dopants or by pointing at ternary oxides or mixed oxides. The final goal is the design of materials with tailored functionality for specific applications through a full understanding of its potentialities and through accurate control at the atomic scale. 
